# Effect of Zinc-Deficient Diet on Oral Tissues and Periodontal Indices in Rats

**Published:** 2014

**Authors:** Seyed Ali Seyedmajidi, Maryam Seyedmajidi, Aliakbar Moghadamnia, Zohreh Khani, Samir Zahedpasha, Niloofar Jenabian, Gholamali Jorsaraei, Sohrab Halalkhor, Mina Motallebnejad

**Affiliations:** 1 *Dental Materials Research Center, Babol University of Medical Sciences, Babol, Iran.*; 2 *Cellular and Molecular Biology Research Center, Babol University of Medical Sciences, Babol, Iran.*; 3 *Students' Research Committee, Babol University of Medical Sciences, Babol, Iran.*; 4 *Dental Research Center, Birjand University of Medical Sciences, Birjand, Iran.*; 5 *Fatemeh-Zahra Infertility and Reproductive Health Research Center, Babol University of Medical Sciences, Babol, Iran.*; 6 *Department of Biochemistry,Faculty of Medicine, Babol University of Medical Sciences, Babol, Iran.*

**Keywords:** Zinc, oral tissue, periodontal indices

## Abstract

Zinc (Zn) as a nutritional factor affects the health of the oral tissues. This study was done for the evaluation of the effects of zinc deficiency on the oral tissues of rats. The study was carried out on 14 male Wistar rats, cessation of lactation on the 24^th^ day after birth. The rats were randomly divided into two groups. Zinc deficient (ZD) diet was used for one group and another group was fed with a zinc-containing (ZC) diet. The alterations of the oral tissues in both groups were evaluated clinically after four weeks. Also the gingival index and periodontal pocket depth were recorded. The measurement of serum zinc level was done by atomic absorption spectrophotometry. The microscopic slides of oral tissue specimen were evaluated quantitatively. The serum zinc level of the ZD rats was lower than the ZC group (p< 0.001). According clinical findings, the gingival index was lower in ZC rat (p=0.001), but there was no significant difference regarding the periodontal pocket depth between two groups (p=0.07). Aphthous ulcer was observed in ZD rats on the floor of the mouth. There was no significant difference regarding the epithelial and keratin thickening between two groups. This study indicated that oral and periodontal health was better in ZC rats than in ZD rats. Aphthous lesions were more prominent in ZD rats. This study confirmed that zinc deficiency may endanger oral and periodo ntal structures.

Zn, an essential trace element for the growth of humans and other animals, has a unique and extensive role in biological processes (1-5).

Normal serum level of Zn is 84-159 µg/dl. Also, the serum level of Zn under 83 µg/dl is considered as Zn deficiency (6).

The current Dietary Reference Intakes (RDAs) for zinc has recommended 11 mg for an adult male and 8 mg for an adult female daily (7).

A prominent feature of Zn deficiency is the broad range of produced pathologies and a spectrum of clinical manifestations, including impaired growth, alopecia, anemia, dwarfism, impaired sexual development, dermatitis, loss of hair, poor appetite, abnormal dark adaptation, delayed wound healing and mental lethargy (3, 4, 8-13). Zn is supposed to be utilized in the management and chemoprevention of cancer (14). Many studies in limited number of cases and animals have been published to determine the effect of topical use of mouthrinse or toothpaste with Zn plus other anti-plaque and anti-gingivitis agents on teeth plaque (15-18).

In 2007, Orbak reported, for the first time, that yhyperkeratinization was more prominent in Zn-deficient rats. They suggested that Zn deficiency is a potential risk factor for oral and periodontal diseases (19).

In 2009, Üçkardeş reported that oral Zn supplementation improved the plaque Index and contributed to the prevention of dental caries in primary school healthy children with low socioeconomic level (17). The general manifestat-ions of Zn deficiency have been reported in a number of studies (4, 12, 20-22), but the effect of Zn deficiency on oral tissue has been described only in a few of studies (8, 19, 23). In this study, we investigated the changes in the oral tissues of Zn-deficient rats.

## Materials and Methods

**Study setting**

This investigation was carried out at the Pharmacology Department of Babol University of Medical Sciences (Babol, Iran). The Animal Ethics Committee of Babol University of Medical Sciences reviewed and approved the experiment protocol. The study setting was similar to the study of Orbak et al. (19). Histopathological examination was evaluated quantitatively and the effect of zinc deficiency on keratin and epithelium thickness was evaluated. The quantitative analysis of keratin and epithelium thickness is considered as more accurate measurement of the histopathological evaluation than qualitative evaluation. In this study, 14 male Wistar rats with cessation of lactation on the 24^th^ day of birth were used. The rats were randomly divided into two equal groups: One group was fed with a Zn-deficient diet (ZD), and another group (control group) was fed with a Zn-containing diet (ZC) (containing 0.056 gr of zinc carbonate in 1 kg of diet). The formulated ZD and ZC diets were identical except for the Zn content. We prepared the zinc deficient diet according to the proposed formula in the study of Orbak et al. (19). The Zn-deficient diet was stored at 4°C in plastic containers and handled with plastic gloves and appropriate tools to avoid contamination. The rats were kept individually in stainless steel cages and maintained at 22–25°C with a 12-h light/dark cycle. They were allowed free access to distilled water. The features of Zn deficiency, including oral lesions, loss of appetite, reduced weight gain, hair loss, and diarrhea, were observed in all ZD rats.

**Clinical evaluation**

Oral manifestations due to Zn deficiency were evaluated at the end of the study. The oral tissues were carefully investigated. The number of oral ulcers, their size and location were recorded. The clinical evaluation consisted of gingival index (Le & Sillness, 1963) scoring, and the measurement of probing pocket depths. The measurements were done in the Pharmacology Department, Babol university of Medical Sciences, by the same investigator. Gingival Index scoring system is as follows: score 0= normal gingival / mucosa around tooth, score 1= mild inflammation, slight change in color, slight edema, no bleeding on probing, score 2= moderate inflammation moderate glazing, redness, bleeding on probing. and score 3= severe inflammation marked redness and hypertrophy, ulceration, tendency to spontaneous bleeding. The gingival index scores were recorded on four tooth surfaces (mesial, distal, buccal, and lingual) for all four anterior teeth.

The numerical scores of the gingival index were obtained according to the formula Per rat = ¼ sum of individual scores/number of anterior teeth present for each rat, and then group score was calculated by adding together the individual scores and dividing the total into the number of rats included. The pocket depths were obtained by measuring the distance from the free gingival margin to the base of the pocket. with a thin wire. (Orthodontic wire 0.5 mil, Dentarum, Germany)

**Atomic absorption spectrophotometry**

The changes of the oral tissue in study groups were recorded at the end of the fourth week on experimental diets. Then, all of the rats were sacrificed after anesthesia with chloroform. Blood samples were taken from auxiliary vessels, centrifuged at 3000 RPM for 5 min. Blood samples were stored in a-20°C temperature. Later, the serum Zn level was measured by atomic absorption spectrophotometry (with wavelength 213.9 nano-meter, dilution 1:10 and sample volume 2.5 after dilution)(Flame type UNICAM 929; ATI-Unicam, Cambridge, UK).

**Histological procedure**

For light microscopy, the tissue samples were fixed by immersion in 10% neutral-buffered formaldehyde for 24 hour, dehydrated in a graded ethanol series, and embedded in paraffin wax. 5 µm thickened paraffined sections were stained with haematoxylin and eosin, and examined using an Olympus BX41 light microscope (Olympus, Tokyo, Japan). To measure the epithelium and keratin thickness, the analysis LS Starter program (OLYMPUS Soft Imaging Solution, Müster, Germany) was used.

**Statistical analysis**

Statistical evaluation was performed using the Student’s t-test for comparing the two groups.

## Results

This investigation was carried out on 14 male rats divided into two groups with seven rats in each group. One Group was fed with a ZD diet, and the other group was fed with a ZC diet. The first observation of appetite reduction, loss of hair, diarrhea, and ulcerations of the skin and mucosa, in ZD rats occurred on the fifth day of the study and continued until the end of the experiment.

**Body weight**

The rats' weight was approximately equal at the beginning of the investigation and there was no statistical difference between them (P=0.62). At the end of the study, body weight was reduced in ZD rats, but was advanced in rats fed with a ZC diet with no significant difference (P=0.09) (Table1).

**Serum level of Zn**

The serum Zn level of the ZD rats was lower than the controls (ZC rats) (P< 0.001) (Table 1).

**Oral Manifestations**

The number and surface area of aphthous ulcer is shown in Table 2. In the present study, aphthous ulcer was observed on the floor of the mouth with a high rate of 33.3% and the greatest average surface area of the ulcers was observed on the ventral side of the tongue (2.20±0.86). Although aphthous ulcer was often seen in the ZD group, it was not found in the ZC group.

**Periodontal features**

The mean gingival index for control group was lower than the ZD group (P = 0.001). Although the mean pocket depth in ZD group was higher than the control group, no significant difference, as regards to pocket depth, was found between the two groups of rats (P=0.07) (Tables 1).

**Histopathological findings**

**Fig. 1:**
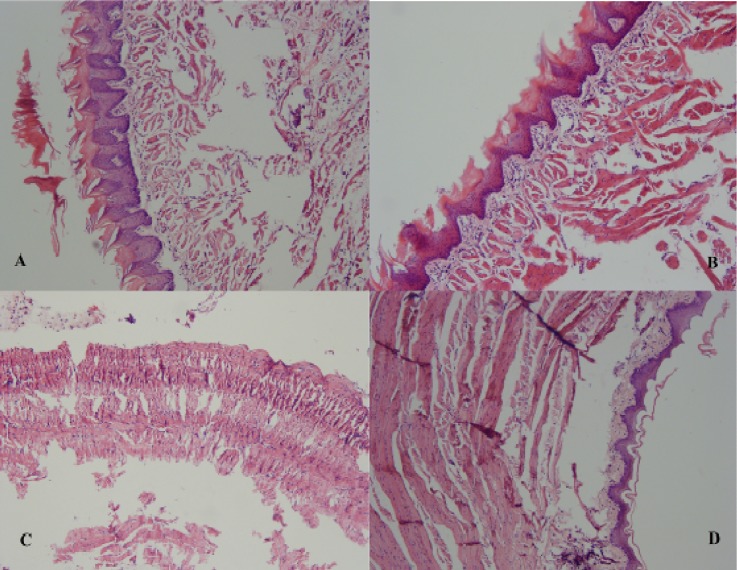
Histopathologic view of the dorsal surface of tongue in (A) ZD group and (B) ZC group. Histopathologic view of the ventral surface of tongue in (C) ZD group indicating ulcer and granulation tissue, and (D) ZC group

**Fig. 2:**
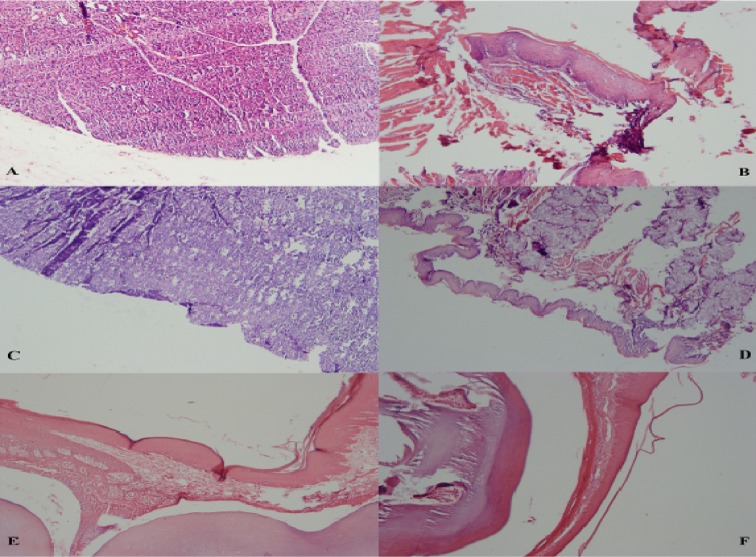
Histopathologic view of the buccal mucosa in (A) ZD group indicating ulcer and granulation tissue, and (B) ZC group and floor of the mouth in (C) ZD group indicating ulcer and granulation tissue, and (D) ZC group. Histopathologic view of the palate in (E) ZD group and (F) ZC group

**Fig. 3:**
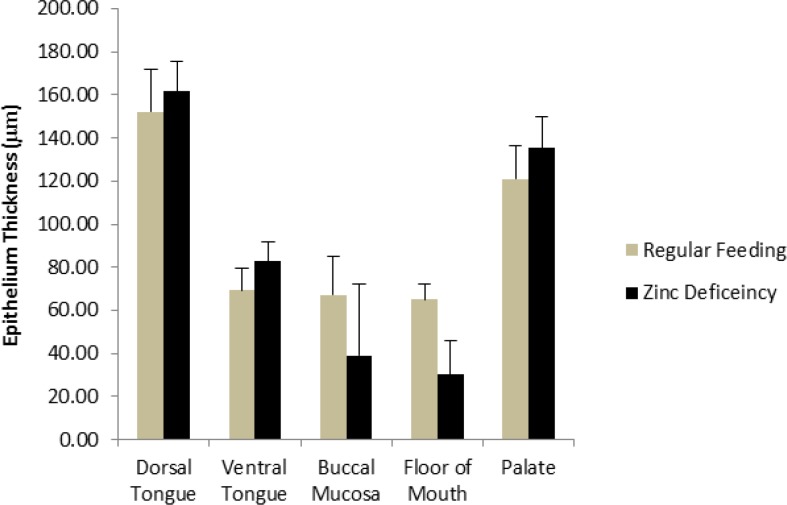
Comparison of epithelium thickness in different parts of mouth in study groups

**Fig. 4:**
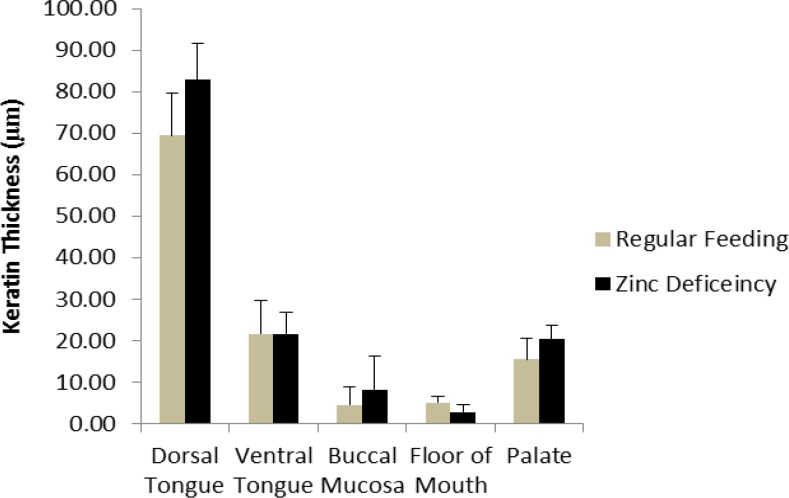
Comparison of keratin thickness in different parts of mouth in study groups

Histological findings were as follows (Figures 1 and 2). Epithelial thickening on the dorsal and ventral surface of the tongue and on the palate in ZD rats was more than the ZC group and on the buccal mucosa and floor of the mouth in ZC group was more than the ZD group. There was no significant difference regarding epithelial thicke-ning (Figure 3). The mean keratin thickness on the dorsal surface of the tongue, buccal mucosa and palate in Zn-deficient rats was more than the ZC group and on the ventral surface of the tongue and floor of the mouth in ZC group was more than the ZD group. There was no significant difference regarding keratin thickness between groups (Figure 4). Hyperkeratinization was much more prominent between the papillae on the dorsal surface of the tongue.

## Discussion

Zn is an important mineral, essential for plants and for normal growth of rats and mice (1). Studies with rats therefore provide a useful model for investigating the effects of dietary Zn deficiency on oral tissues (19).

**Table 1:** Comparison of weight (gr), serum zinc level, gingival index and periodontal pocket depth of rats in ZD and ZC groups at the end of the study

**Groups**	**Number**	**Weight**		**Serum zinc level** **(ppm)**		**Gingival index**		**Periodontal pocket depth**			
		Mean±SD	P value	Mean±SD	P value	Mean±SD	P value	Mean±SD	P value
ZD Group	7	104.73±18.32	0.09	0.02±0.036	<0.001	1.57±0.76	0.001	0.42±0.31	0.07		
ZC Group	7	119.45±10.45	0.09	0.33±0.097	<0.001	0.50±0.76	0.001	0.21±0.18	0.07	

**Table 2:** The number and mean ± SD of surface area of minor aphthous ulcers in group I rats

Group I	Dorsal surface of tongue	Ventral surface of tongue	Buccal-labial mucosa	Floor of the mouth	palate
Number of ulcers	1	5	5	6	1
Surface area of ulcers	1.57±0.00	2.20±0.86	1.88±0.7	1.83±0.64	1.17±0.00

Only a few studies presented oral manifestations in rats with ZD diet (10, 19). In addition, it has been reported that Zn deficiency might produce marked effects on about all components of the immune system (22, 24). The study protocol was similar to that Orbak et al. (19) Our findings of growth retardation in Zn-deficient rats and growth acceleration in rats receiving supplementary Zn have confirmed that Zn is an important element for growth which was also indicated in the study of Orbak et al.

The diagnosis of Zn deficiency can be confirmed by both clinical features and laboratory findings (3, 12, 25). Previous studies evaluated Zn concentrations in serum using atomic absorption spectrophotometry (19, 25, 26). In our study, we also used this method and identified that the serum Zn level of ZD rats was lower than the rats with ZC diet. Zn is essential in both cell-mediated and homoral immunity. Recurrent aphthous stomatitis (RAS) is one of the most common ulcerative lesions of the oral cavity all around the world. It seems that aphthous ulcers have a multifactorial etiology. Also, recent articles have suggested that patients with aphthous ulcers may have primary immune abnormalities or immune deficiency (10-11, 24, 27). In the study of Khademi et al., it was found that serum Zn level is significantly lower in Recurrent Aphthous Stomatitis (RAS) patients, thus the determination of serum Zn level, and prescribing Zn if its serum level is low, are recommended in RAS patients (28). In this study, we investigated the changes associated with Zn deficiency in the oral tissues.

Aphthous ulcer was often seen in the ZD group, on the floor of the mouth with a high rate of 33.3% in contrast to Orbak et al.'s study which was observed on the alveolar mucosa with a high rate of 29.9%.

In our study, a statistically significant difference in gingival index values was found between the ZD group and the ZC group which was also similar to the Orbak et al.'s study (19). One of the most commonly used clinical parameters in the diagnosis and prognosis of periodontal diseases is the periodontal pockets. Similar to Orbak et al.'s study, in our study, periodontal pocket depth was also increased. However, no significant difference of pocket depth values between the two groups was found. Rats and mice fed with ZD diet develop parakeratosis of normally orthokeratinized oral mucosa (29). In our experiment, epithelial thickening on the dorsal and ventral surface of the tongue and on the palate in ZD rats was more than ZC group. There was no significant difference regarding keratin thickness between groups. Hyperkeratinization was much more prominent between the papillae on the dorsal surface of the tongue However, in Orbak et al.'s study, this hyperkeratosis was found on the dorsal surface of the tongue in ZD rats (19).

According to the results of this study, ZC rats had a better oral health than ZD rats. Hence, ZD diet may endanger the health and structure of oral and periodontal tissues.
